# Impact of Actual Use Behavior of Healthcare Wearable Devices on Quality of Life: A Cross-Sectional Survey of People with Dementia and Their Caregivers in Ghana

**DOI:** 10.3390/healthcare10020275

**Published:** 2022-01-30

**Authors:** Ebenezer Larnyo, Baozhen Dai, Abigail Larnyo, Jonathan Aseye Nutakor, Sabina Ampon-Wireko, Edmund Nana Kwame Nkrumah, Ruth Appiah

**Affiliations:** 1 Department of Health Policy and Management, School of Management, Jiangsu University, 301 Xuefu Road, Zhenjiang 212013, China; dr.ebenlarnyo@ujs.edu.cn (E.L.); jnutakor@gmail.com (J.A.N.); amponwirekosabina@gmail.com (S.A.-W.); ruthapp27@yahoo.com (R.A.); 2 School of Management, Jiangsu University, 301 Xuefu Road, Zhenjiang 212013, China; abigaillarnyo@hotmail.com (A.L.); nkrunak@gmail.com (E.N.K.N.)

**Keywords:** dementia, healthcare wearable devices, extended UTAUT, actual use behavior, well-being, quality of life, SF-36, Ghana

## Abstract

The increasing prevalence of dementia has necessitated the introduction and use of healthcare wearable devices to augment the healthcare services delivered to people with dementia (PWDs). Unfortunately, evidence on user feedback and the real impact of the actual use of these devices on the quality of life of PWDs has not been fully explored, especially in the context of developing countries. This study, therefore, seeks to evaluate the impact of actual use behavior (AUB) of healthcare wearable devices on the quality of life (QoL) of PWDs in Ghana by using constructs from the extended Unified Theory of Acceptance and Use of Technology (UTAUT2) model and the SF-36 quality of life instrument. Data were collected from people with dementia and, in some cases, caregivers (acting as proxies) in three administrative regions of Ghana using structured questionnaires. Of the 420 questionnaires distributed, 356 were returned, giving a response rate of (356/420, 84.8%). Three hundred and twenty (320) responses were used for further analysis, employing the Partial Least Squares based on Structural Equation Modeling. The results showed a significant positive relationship between AUB and QoL (*p* < 0.001). Other predictors such as behavioral intention, facilitating conditions, effort expectancy and social influence were also found to significantly impact the actual use behavior of healthcare wearable devices among PWDs. The study also revealed that 4% (14) of patients rated their reported health transition associated with the use of healthcare wearable devices as excellent, 42.5% (136) as very good, and 39.4% (126) as good, respectively at the time of the study compared to a year ago. This study provided empirical evidence of the positive impact of the actual use of healthcare wearable devices on the quality of life of PWDs and further highlighted the factors that drive actual use behavior among PWDs in Ghana. Based on the findings of this study, stakeholders need to create the necessary conditions that will facilitate the adoption and use of healthcare wearable devices, as this will help improve the quality of life, reduce the burden of the disease on caregivers, and enable independent living of PWDs.

## 1. Introduction

The global statistics on health as reported by World Health Organization (WHO) suggest that over 55 million people have dementia, with approximately 60% living in low- and middle-income nations [1]. The report further revealed that an estimated 10 million new persons are diagnosed with dementia yearly. In developing countries, the prevalence of dementia among individuals has seen an astronomic increase over the past years. Data available on dementia cases in sub-Saharan Africa revealed about 2.13 million patients with dementia in the region as of 2015 [2], representing over 367,000 new cases in a year. Within the next 20 years, the number of dementia cases is expected to double, reaching 3.48 million in 2030 and 7.62 million in 2050 [3,4].

According to the latest data published in 2017 by the WHO, Alzheimer’s and dementia-related deaths in Ghana reached 1701 (0.8%) of total deaths, with an age-adjusted death rate of 16.47 per 100,000 [4], putting the country at number 102 in the world in terms of dementia cases [4].

As established by previous studies, dementia impacts different aspects of a person’s life and has been identified as a significant cause of disability and dependency among the elderly [5]. More so, apart from compromising the living standards of the people diagnosed with the disease, it also affects the physical, psychological, social, and economic well-being of patients and that of the caregivers, families, and society as a whole [6]. In the absence of a cure and effective treatment, the primary goal in dementia care is to ensure optimization of well-being, identify and manage the illness, and facilitate effective treatment of behavioral and psychological symptoms by guiding caregivers [1,5]. As estimated, if the population ages over the next several decades, dementia cases and their associated costs will dramatically increase. Hence the need for an increase in the quality and variety of healthcare services offered to these patients [1]. 

Unarguably, the introduction of wearable technologies in the healthcare sector has brought about tremendous change in personalized healthcare service delivery. Wearable technology consists of clothing and accessories that incorporate advanced technologies to assist individuals wearing them to perform their daily tasks quickly and efficiently. Such technologies are very fundamental to monitoring the physiological data of older people or individuals with chronic conditions and facilitating timely clinical interventions.

In the health sector, patients now have access to the best diagnostic tools, new and cutting-edge treatments, and a myriad of minimally invasive procedures resulting in less pain and quicker healing. Not only do patients have access to quick and accurate medical information using their wearable devices, but they also have the option to use apps to monitor doctor’s appointments and receive reminders for medications. These devices collect crucial information both continuously and noninvasively in real time regarding a wearer’s health [7]. Specifically, the rise in internet usage and technological familiarity among the global population has also enhanced the use of these devices, even among developing countries. It is, therefore, not surprising that much research and investments have been made to develop technologies, such as the sensor-based networks for activity monitoring, fall and wandering detection, smart socks, clever care smartwatches, and various eHealth applications to support patients suffering from illnesses associated with aging, such as dementia, in order to improve their quality of life [4,8,9,10,11]. Furthermore, projects such as the MARIO Project 2018, have succeeded in developing robots that remind users to perform everyday tasks, such as eating, drinking, and when to go shopping, as well as social events, such as family birthdays and anniversaries [12]. These robots have been found to be very useful and preferred by PWDs [12]. Other projects, such as SMART4MD aimed at developing a tool to help PWDs to stick to their treatment regimens and exchange data with their caregivers and physicians, as well as for caregivers to better monitor PWDs and share their health information with their doctors, and the CAREGIVERSPRO-MMD Project aimed at developing a social platform that assists PWDs with chronic conditions by reducing the frequency with which they attend care facilities by employing self-management tools/systems and thereby improving their everyday activities, have been carried out and have all received great acceptability by PWDs [13]. 

Due to issues of cognitive limitations and other risk factors associated with aging, studies have suggested that elderly users, especially PWDs exhibit resistance to the use of healthcare devices [2,13]. Notwithstanding the existence of these studies, other research and projects have demonstrated that, with the right approach to design considerations, this resistance could be reduced tremendously, leading to the adoption of wearable devices by PWDs [14,15,16,17,18,19]. For instance, studies have revealed that GPS-based wireless tracking devices, such as GPS Smart Sole, Freedom GPS Locator Watch, Safe-link, Mindme Locate, MXLOCareTM, iTraq3, and PocketFinder+, and others, such as Fitbit, Mi band, Oura ring, and Alice PDx, have been preferred and widely used by PWDs [20]. The wearable lullaby, a non-ambulatory device designed for PWDs to prevent them from waking up during the night and thereby disturbing the sleep of their caregivers, has also been introduced and adopted by several PWDs [21].

Amidst all these technological advancements in personalized healthcare delivery [22,23], the primary concern and expected outcome is how these technologies improve the quality of life (QoL) of PWDs. The WHO defined QoL as “individuals’” perception of their position in life in the context of the culture and value systems in which they live and their goals, expectations, standards, and concerns [24]. These perceptions are assessed using an individual’s physical and mental health, education, recreation and leisure time, social belonging, wealth, employment, and the environment. The Center for Disease Control and Prevention (CDC) further expanded the concept to encompass all aspects of QoL that can affect physical and mental health perceptions (e.g., energy level, mood), health risks and conditions, functional status, social support, and socioeconomic status [25,26,27]. QoL is of significant consequence across the whole spectrum of the human health continuum. Studies have indicated that evaluating an individual’s QoL sheds light on the severity of their illnesses and could help provide salient information on planning interventions that may improve their overall quality of life [27,28]. 

Literature has contributed to understanding the quality of life of PWDs in its generic form; however, most of these studies have focused on the relationship between quality of life and objective indicators, such as income levels and social status of PWDs [28,29,30]. In contrast, research employing subjective indicators relating to the quality of life or well-being regarding the use of healthcare wearable devices remains, to a great extent, in abeyance, especially in developing countries. Furthermore, studies on wearable devices in the health industry have gained significant attention among developed countries, yet the same cannot be said for developing countries, especially countries in sub-Saharan Africa. This phenomenon has the tendency to lead to the exclusion of user experience of a vital demographic, hence skewing design considerations of healthcare wearable devices to only users from developed countries. Since user experience among device users in developed countries may differ from those in developing countries due to variation in socioeconomic determinants in these countries, it is imperative to understand these experiences in the context of developing countries, such as Ghana. Hence, to address these vital concerns, this current study intends to:

(i). Assess the impact of the actual use of healthcare wearable devices on the quality of life (QoL) of PWDs, using the extended UTAUT model and SF-36 quality of life instruments.
(ii). Provide an overview of the determinants of actual use behaviors among PWDs by assessing the influence of factors, such as behavioral intention, effort expectancy, social influence, facilitating conditions, resistance to change, and technology anxiety, on the actual use behavior of healthcare wearable devices among PWDs.

## 2. Theoretical Basis and Conceptual Framework

### 2.1. Extended UTAUT Model

The theoretical basis for this study is founded and built on the concept of the Extended unified theory of acceptance and use of technology (UTAUT). Unlike the UTAUT model, which explains user intentions to use an information system and subsequent usage behavior, several other studies have employed the extended UTAUT model to explain their hypothesis on different modes and mediums of technological acceptance among individuals [2,31,32]. Some of these studies have extended the model to assess the influence of online social support on network information technology usage [33], to evaluate individual adoption of social networks in organizations [34], and to study the determinants of acceptance of mobile learning in other jurisdictions [35,36,37,38]. 

Conceptually, the extended UTAUT consists of four antecedents of the traditional UTAUT model: performance expectancy, effort expectancy, social influence, and facilitating condition, which act as determinants of actual use behavior and behavioral intention to adopt technology such as healthcare wearable devices [2]. In this study, an examination of each dimension of the extended UTAUT model was explored to understand the research issue. 

#### 2.1.1. Performance Expectancy (PE)

According to the model’s proponents, the performance expectancy construct helps assess how individuals believe that using a particular system will help them attain the desired goals in their job performance [38,39,40]. Using this construct as part of the tools to determine the actual use behavior will be very beneficial, as it gauges the perceived usefulness of healthcare wearable devices. Thus, we posit that;

**Hypothesis** **1** **(H1).** *PE has a positive impact on the dementia patient’s behavioral intention to use healthcare wearable devices.*

#### 2.1.2. Effort Expectancy (EE)

Derived from the perceived ease of use (PEOU) component of TAM, effort expectancy, an essential construct in assessing users’ intention, is defined as “the degree of ease associated with the use of the system” [39,40,41]. For any system to be used effectively to meet the needs of its users, the degree of perceived difficulty associated with the use of such a system is critical. Thus, it is imperative to determine how users perceive the degree of difficulty related to the use of healthcare wearable devices.

**Hypothesis** **2** **(H2).** *EE positively impacts the dementia patient’s behavioral intention to use healthcare wearable devices.*

#### 2.1.3. Social Influence (SI)

Another essential construct in the extended UTAUT model is the social influence (SI) construct. According to Venkatesh et al., 2003, SI is used to determine how strongly end users of a system perceive that others—family, friends, and society—believe they should use a system [31,39]. In highly collective countries where a sense of community is embedded into the socialization process of citizens, the opinions and advice of family members, friends, and society are considered relevant and play a significant role. These opinions ultimately influence the decision-making process of whether or not to adopt healthcare wearable devices, consequently affecting the quality of life of dementia patients. Previous studies have shown that the behavior of others in a user’s social and work circles significantly impacts that end user’s use of technology [42,43,44].

Thus, it is crucial to evaluate social influence’s contributing role in actual use behavior and, consequently, quality of life. Therefore, this study posits that:

**Hypothesis** **3** **(H3).** *SI positively impacts the dementia patient’s behavioral intention to use healthcare wearable* *devices.*

#### 2.1.4. Facilitating Conditions (FC)

Facilitating conditions in the extended UTAUT is how individuals perceive that institutional and technical facilities exist to augment their system use [39,45]. This construct comprises training and support, shared belief in the system, and project communication. Research has revealed that facilitating conditions, such as an individual’s intellectual and cognitive abilities and the perceived cost of learning new technologies, affect the actual use behavior of technology [2,13,46]. Thus, this study examines four key areas regarding the effect of facilitating conditions on behavioral intention and actual use behavior of healthcare wearable devices. This construct will help assess the availability of the necessary resources, knowledge, adequacy of the essential expertise possessed by medical practitioners to allow patients to use healthcare wearable devices, and, finally, the compatibility of the healthcare wearable devices with other technologies PWDs use.

**Hypothesis** **4** **(H4).** *FC positively impacts the dementia patient’s behavioral intention to use healthcare wearable devices.*

**Hypothesis** **5** **(H5).** *FC positively impacts the dementia patient’s actual use behavior of healthcare wearable devices.*

#### 2.1.5. Behavioral Intention (BI)

There is an evidenced relationship between behavioral intention (BI) and actual use behavior (AUB) in many fields, which indicates BI is a valid predictor of actual use behavior [47,48,49]. Empirically, the behavioral intention construct has been tested and found to be able to explain the user’s actual use behavior of technology [39]. Research by Han et al. 2004 on determining physicians’ behavior intentions regarding mobile technology use revealed that actual use behavior (AUB) influences the behavioral intention of physicians in their quest to use mobile health technology [50]. Adopting this construct will help uncover its contributing role to actual use behavior and how it helps achieve the quality of life among patients with dementia who use healthcare wearable devices. This study posits that:

**Hypothesis** **6a** **(H6a).** *BI positively impacts the dementia patient’s actual use behavior of healthcare wearable devices.*

**Hypothesis** **6b** **(H6b).** *BI has a partial mediating effect on FC and AUB.*

#### 2.1.6. Technology Anxiety (TA) and Resistance to Change (RC)

Researchers have found that technological anxiety and resistance to change impact geriatric technology uptake [51]. Technology anxiety is a negative affective, emotional response or dread of discomfort that patients experience due to utilizing technology [2,52]. As a result, it is vital to investigate the effects of those anxieties on adoption behavior. Furthermore, it has been discovered that resistance to change reduces the desire to use technology [2,53]. It is also asserted that older persons are resistant to change [13,54]. Because dementia is usually connected with aging, TA and RC will help put these assumptions to the test. The study, therefore, proposes the following hypotheses:

**Hypothesis** **7** **(H7).** *RC has a negative impact on the dementia patient’s behavioral intention to use healthcare wearable devices.*

**Hypothesis** **8** **(H8).** *TA has a negative impact on the dementia patient’s behavioral intention to use healthcare wearable devices.*

#### 2.1.7. SF-36

This study adopted the SF-36 to measure the subjective quality of life of PWDs associated with healthcare wearable device use [55]. The SF-36 instrument consists of eight quality-of-life dimensions: physical functioning, physical roles, emotional roles, social functioning, bodily pain, mental health, general health, and vitality [55]. Since dementia first and foremost affects cognition and, subsequently, affects patients’ general health, this study categorized the eight QoL dimensions into two broad aspects of health, that is, general health and mental health. The hypothesis below was posited to evaluate the impact of actual use behavior on quality of life:

**Hypothesis** **9** **(H9).** *AUB of healthcare wearable devices has a positive impact on QoL.*

#### 2.1.8. Moderating Effect

TA and RC were used as moderating constructs to test their significance on the actual use behavior and their contributing roles on the quality of life [1,35] as posited below:

**Hypothesis** **10a** **(H10a).** *RC will negatively moderate the effect of effort expectancy on behavioral intention to use healthcare wearable devices.*

**Hypothesis** **10b** **(H10b).** *RC will negatively moderate the effect of social influence on behavioral intention to use healthcare wearable devices.*

**Hypothesis** **10c** **(H10c).** *RC will negatively moderate the effect of facilitating conditions on behavioral intention to use healthcare wearable devices.*

**Hypothesis** **11a** **(H11a).** *TA will negatively moderate the effect of effort expectancy on behavioral intention to use healthcare wearable devices.*

**Hypothesis** **11b** **(H11b).** *TA will negatively moderate the effect of social influence on behavioral intention to use healthcare wearable devices.*

**Hypothesis** **11c** **(H11c).** *TA will negatively moderate the effect of facilitating conditions on behavioral intention to use healthcare wearable devices.*

#### 2.1.9. Conceptual Framework

**Figure 1:**
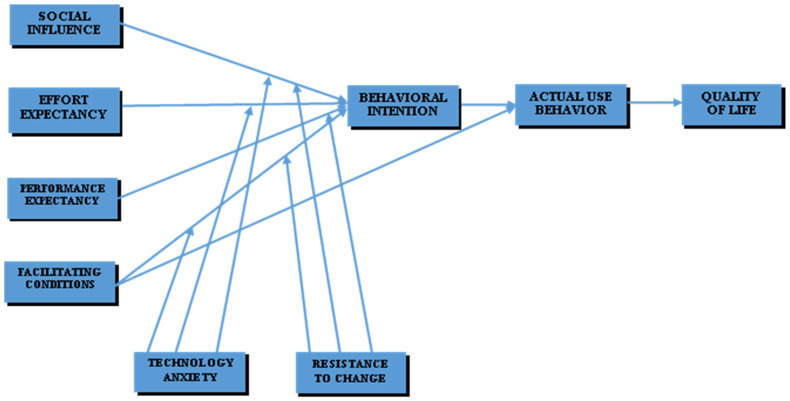
Conceptual framework.

A conceptual framework was developed based on an extant literature review and the hypothesis posited, as shown in Figure 1.

## 3. Methodology

### 3.1. Research and Questionnaire Design, and Data Collection

With the aid of structured questionnaires adapted from existing literature (see Appendix A Table A1), this study collected data from people with dementia who were living in their homes and, in some cases, their familial caregivers (acting as proxies) for those with severe cognitive impairment. Respondents were recruited from three cities in three administrative regions: Accra, Kumasi, and Koforidua in the Greater Accra, Ashanti, and Eastern regions of Ghana, respectively, from June 2019 to December 2019. The recruitment of respondents for the study was solely based on the availability and willingness of familial caregivers and PWDs to participate in the survey [2]. The Mini-Mental Status Examination (MMSE), a clinical tool for the diagnosis of dementia and other neurological degenerative diseases, was used in recruiting people with dementia [56]. MMSE items were scored between 0 and 30, with a maximum score of 30. These scores were grouped into four categories, with 25 to 30 depicting PWDs with a questionably significant degree of impairment, 20 to 25 as mild, 10 to 20 as moderate, and 0 to 10 as a severe degree of impairment [56]. 

To obtain reliable and reproducible results, inclusion and exclusion criteria were set. In order for a respondent to qualify to participate in this study, a PWD must be aged 50 or older, while a caregiver must be aged 18 or older, and the PWD must be living independently and not be in a care facility. Further, the PWD must have a Mini-Mental Status Examination (MMSE) [56] severity score of between 0 and 25 to qualify to participate. If the MMSE score is between 0 and 10, the participant is considered to have severe impairment and, thus, there must be a proxy (strictly a family member who is a caregiver) present to respond on the PWD’s behalf. Moreover, the respondent must be able to understand and agree to participate voluntarily in the study by affirming the informed consent statement in order to be included in the study. Because this study aims to evaluate the impact of actual use behavior on quality of life, PWDs had to be currently using or had to have been using a healthcare wearable device in the last one year in order to meet the inclusion criteria. Conversely, PWDs below 50 years of age and caregivers below 18 years of age were excluded, while PWDs with MMSE scores of less than 10 who had no caregivers or proxies immediately available and present were also excluded from the study. Respondents who did not affirm the informed consent statement for participation were also excluded from the study. 

The questionnaire for this study was developed in English using a 5-point Likert scale ranging from (1) “strongly agree” to (5) “strongly disagree” for items of the extended UTAUT constructs and the different scales for the constructs of the SF-36 items, as proposed by [3] (see Appendix A Table A1). Translators were recruited to translate the questionnaire items from English to the various local dialects depending on the location of the respondents who could not understand or speak English. Respondents who could read and write were required to complete the questionnaire without the assistance of an intermediary or translator.

Out of the 420 questionnaires distributed, 356 were returned, giving a response rate of 356/420, 84.8%, which is considered good. Of the total 356 responses received, 36 (10%) were excluded due to substantially incomplete responses or missing values, leaving 320 for further analysis.

### 3.2. 36-Item Short-Form Survey (SF-36) Coding and Scoring

The SF-36 coding and scoring algorithms developed by Ware et al., 1993 were adopted without changing the original algorithm [57] to provide standardization of content and scoring for the instruments used to measure the quality of life associated with healthcare wearable device use. The scales were computed so that a high score indicates better quality of life of patients.

### 3.3. Quality Control Measures and Handling Biases

To prevent deviations from the study protocol and avoid errors during data collection [58,59], quality control and bias handling measures were implemented. The targets of the quality control procedures were the people involved in data assessment used for the different measurement procedures. A total of 14 officers, consisting of seven interviewers/enumerators and seven translators, were trained for the baseline assessment in the data collection sites. The training was conducted by the principal researcher with the aim of equipping the research team with the requisite procedures and instructions needed for the data collection and ensuring the enumerator could adapt to changes in the field as and when necessary.

Enumerators were asked to allow respondents who could answer the questionnaires themselves to fill out the hard copy of the questions with supervision. After the self-administered questionnaires were completed and handed in, the data enumerators submitted the completed questionnaires to the field leader so the data could be checked for completeness and errors. Missing or inconsistent information caused by either respondents or enumerators was immediately identified and cross-checked with the enumerators. That missing or inconsistent information was further verified by consulting the respondents, resulting in complete and logically consistent datasets from each respondent.

Additionally, the SF-36 questionnaire, which concentrated on the respondents’ experiences, feelings, beliefs, perceptions, and convictions concerning their health-related quality of life, consisted of closed-ended structured questions. These questions related specifically to eight quality-of-life indicators and two summary measures that revolved around both physical and mental health. The closed-ended questions of the SF-36 questionnaire compelled respondents to select their responses from a set of possible answers compiled by Ware Jr and Sherbourne 1992 [55]. This resulted in the elimination and, in some cases, reduction of errors that would have occurred as a result of asking respondents to provide their own formulated responses. Moreover, all other questions relating to actual use behavior, with the exception of the demographic information and MMSE, were closed-ended. These questions complied with the methodological guidelines for closed-ended questions [60,61].

### 3.4. Data Analysis

The partial least squares (PLS) method, a statistical analysis technique based on the SEM, was used to test and validate the proposed model and the relationships among the hypothesized constructs due to its robustness of estimations and statistical power [2,62,63], with the help of SmartPLS 3.0. The validation of the model was carried out by evaluating the measurement and structural models.

Internal consistency reliability, convergent validity, and discriminant validity were used to assess the measurement model. This was carried out using the outer loadings, average of variance extracted (AVE), composite reliability (CR), Cronbach alpha (α), the Fornell–Larcker criterion, Heterotrait–Monotrait ratio of correlation (HTMT), and cross loadings [63].

The evaluation of the structural model was conducted using the collinearity assessment, path coefficient *(β)* using t-statistics, coefficients of determination (R^2^ values), effect size (*f*^2^ values), predictive relevance (Q^2^ values) obtained using blindfolding, and effect size (q^2^ values).

## 4. Results

### 4.1. Descriptive Statistics

Frequencies and percentages were calculated for the demographic variables: age (years), gender, marital status, level of education, and years of illness or caregiving split by respondents, as presented in Table 1.

Table 2 shows the reliability, central tendency, and variability of the SF-36 scales used to solicit responses from patients and proxies. Most of the Cronbach alpha values are greater than or equal to 0.70 (α ≥ 0.7), indicating a high internal consistency for the items [64], except general health and role emotion, which had α *<* 0.7.

### 4.2. Evaluation of Measurement Model

**Figure 2:**
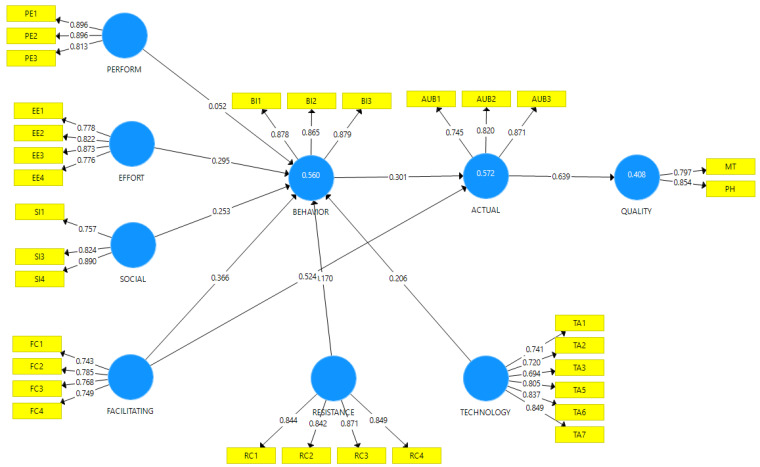
Diagram indicating the various latent and observed variables and their loadings.

The outer loadings, internal consistency reliability, convergent validity, and discriminant validity were examined for the measurement model [64,65], as presented in Table 3. The outer loadings for all constructs showed values greater than the acceptable threshold of 0.70, except TA3, which had an outer loading value of 0.694. Moreover, composite reliability and Cronbach alpha (α) values for all the constructs were greater than 0.70, suggesting strong internal consistency reliability [66]. The convergent validity was assessed by an average variance extracted (AVE) and the factor loadings of the constructs. AVE values were all above the recommended level of 0.50 [65], as shown in Table 3. Figure 2 shows the various constructs with their respective loadings.

The Fornell–Larcker criterion, Heterotrait–Monotrait ratio of correlation (HTMT), and cross loadings were used to evaluate the discriminant validity of the model. As shown in Appendix A Table A3, the square root of the AVE of the constructs was mostly greater than their correlation with other constructs, and the diagonal items were larger than the entries in corresponding columns and rows, hence satisfying the discriminant validity criteria [65,67], except for FC→EE and TA→RC. Results of HTMT revealed that almost all the constructs had HTMT values less than 1, except for FC→EE (HTMT = 1.073), QoL→BI (HTMT = 1.178), and QoL→FC (HTMT = 1.276), which had HTMT values greater than 1. Finally, all loadings of the indicators were greater than their associated constructs, except TA2 and TA3; therefore, cross-loading criteria were fulfilled, as shown in Appendix A Table A4.

### 4.3. Evaluation of Structural Model

The collinearity assessment (variance inflated factor (VIF)), path coefficient (*β*), t-statistics, coefficients of determination (R^2^ values), blindfolding and predictive relevance (Q^2^ values), and effect size (q^2^ values) were used to evaluate the structural model. VIF shows all the latent variables have a VIF less than 5; therefore, the model has no collinearity problem [68], as shown in Appendix A Table A5.

The hypothesized relationship between constructs was assessed using the path coefficient *(β)*. All the constructs had a strong positive relationship, except for RC→BI, which had −0.149, indicating a weaker relationship between the two constructs. As shown in Table 4, *t*-values for all the relationships between the constructs were significant at 5 and 10 per cent, respectively [69,70]. All the *p*-values of the relationship between the various constructs were significant at *p* < 0.001 or *p* < 0.05, except for PE → BI, which had *p* = 0.25, as reported in Table 4.

R^2^ values of 0.572, 0.560, and 0.408 for AUB, BI, and QoL, respectively, indicate that the model has moderate predictive accuracy. Moreover, Q^2^ values of 0.370, 0.407, and 0.271 for AUB, BI, and QoL are greater than zero (0), suggesting the model has predictive relevance for the endogenous construct. The q^2^ value was used to assess the exogenous construct’s contribution to the endogenous latent variable’s Q^2^ value. Based on construct cross-validated redundancy results, the effect size (q^2^) for AUB→QoL was 0.157, indicating a medium effect, and that for BI→AUB was 0.062, indicative of a small effect size [68,71].

#### Hypotheses Testing

Results from Table 4 showed EE→BI (t-statistics = 4.410, *β* = 0.295, *p* < 0.001), SI→BI (t-statistics = 4.929, *β* = 0.253, *p* < 0.001), FC→BI (t-statistics = 5.383, *β* = 0.366, *p* < 0.001), FC→AUB (t statistics = 10.351, *β* = 0.524, *p* < 0.001), BI→AUB (t-statistics = 5.666, *β* = 0.301, *p* < 0.001), RC→BI (t-statistics = 2.136, *β* = −0.170, *p* < 0.05), TA→BI (t statistics = 2.912, *β =* 0.206, *p* < 0.001), and AUB→QoL (t statistics = 17.448, *β =* 0.639, *p* < 0.001) were significant. Thus, Hypotheses 2, 3, 4, 5, 6a, 7, 8, and 9 were supported. However, PE→BI (t-statistics = 1.145, *β =* 0.052, *p* = 0.25) was insignificant, thus not supporting Hypothesis 1.

### 4.4. Evaluating the Mediating Role of BI on FC and AUB

The result of mediation shows an indirect effect of 0.110 significant effect for FC→AUB (*p* < 0.001), as shown in Table 5. Additionally, since the indirect effect is significant and the direct effect is significant, we conclude a partial mediation exists of BI on FC and AUB, thus satisfying Hypothesis H6b [71,72].

### 4.5. Evaluating Moderatiing Effects of TA and RC on BI

The moderating effects were examined for H10a, H10b, H10c, H11a, H11b, and H11c, respectively, as shown in Table 6 below. Regarding the moderating effect of resistance to change, RC × EE→BI and RC × FC→BI were insignificant, thus, not supporting H10a and H10c. However, the moderating effect of RC × SI→BI was significant, therefore supporting H10b.

The sign of the moderating effects showed positive moderation for the effect of effort expectancy on behavioral intention in terms of resistance to change. This result contradicts the hypothesis that resistance to change would negatively moderate the impact of effort expectancy, facilitating conditions, and social influence on behavioral intention to use healthcare wearable devices.

The evaluation of the moderating effect of technology anxiety on effort expectancy, facilitating conditions, and social influence on behavioral intention was insignificant, thus not supporting H11a, H11b, and H11c. However, the signs of the moderating effects showed TA × EE→BI had a negative path coefficient (β) of −0.052, which supported the assertion that technology anxiety will negatively moderate the effect of effort expectancy on behavioral intention to use healthcare wearable devices.

## 5. Discussion

This study applied the constructs of the extended UTAUT model: performance expectancy (PE), effort expectancy (EE), social influence (SI), technology anxiety (TA), facilitating conditions (FC), and resistance to change (RC), and the SF-36 measuring items, to evaluate the impact of actual use behavior of healthcare wearable devices on the quality of life of dementia patients. In line with the overall objective of this study, it was posited that the actual use behavior (AUB) of healthcare wearable devices has a positive impact on quality of life (QoL). Based on the results obtained, this study found this claim to be true. Results revealed AUB→QoL had a t-statistic = 17.448, with a path coefficient (*β*) = 0.639 and *p* < 0.001, making it the highest predictor in the research model. The use of wearable technologies has proven to be very useful in augmenting the independent living of dementia patients. Research has also shown evidence of such instances where such technologies were able to serve as a source of entertainment and a means of engaging with PWDs [73,74], making it easier for them to stay out of institutional homes, thereby relieving the burden of caregiving [75,76,77]. Healthcare wearable technologies have served as useful support tools, as they have demonstrated the ability to improve communication between caregivers and PWDs [75,78] and, in some instances, they have helped to enhance the interactions of patients with their friends and social networks [79]. In terms of the actual responses to the eight items of the SF-36 instrument used to determine the impact of actual use behavior of healthcare wearable devices on the physical functioning (the extent to which the respondents’ perceptions of their quality of life are influenced by their physical conditions), physical role, bodily pain, general health, vitality, emotional role, mental health, social functioning and the reported health transition, this study found a very high degree of perception of quality of life with regards to healthcare wearable device use. When asked how they (respondents) perceived their health compared to a year ago after using healthcare wearable devices, 4.4% of respondents rated their reported health transition to be excellent, 42.3% very good, 39.5% good, 9.4% rated fair, and only 4.4% of respondents rated their reported health transition since using healthcare wearable devices as poor.

In the quest to assess the physical functioning of PWDs regarding the use of healthcare wearable devices, the majority (83.1%) of respondents revealed that healthcare wearable devices did not limit them in doing vigorous activities, but rather augmented their vigorous activities. Healthcare wearable devices were also found not to limit patients in doing moderate activities, nor did it limit them in lifting, carrying groceries, climbing one or several flights of stairs, bending, kneeling, stooping, or in walking more than a mile or even several hundred yards, suggesting that their physical functioning was rather improved through the use of healthcare wearable devices. Similar trends were also found in relation to physical role, bodily pain, general health, vitality, emotional role, and mental health, as shown in Appendix A Table A2.

Results of this study supported the posited hypotheses with regards to effort expectancy, social influence (SI), facilitating conditions (FC), behavioral intention (BI), resistance to change (RC), and technology anxiety (TA). In our previous study on the factors that influence the adoption of wearable devices among PWDs using family caregivers as proxies, it was found that a significant relationship existed between social influence and behavior intention of PWDs to adopt wearable devices [2]. A study conducted by Hoque and Sorwar, 2017 on the adoption of mHealth among the elderly in Bangladesh also observed that social influence (SI) and behavioral intention (BI) have a significant influence on the adoption and usage of mHealth [13]. These findings are consistent with our current study results. In a highly collective country such as Ghana, where a sense of community is embedded into the socialization process of families, the opinions and advice of other members of society and family are considered relevant and play a significant role in the decision-making process of whether or not to use healthcare wearable devices [4]. For instance, the four questions posed to determine to what extent patients and caregivers perceived the society influences their decision to use or recommend healthcare wearable devices in this study revealed that the majority of respondents agreed to the notion that society plays a part in their decision-making process. Thus, device manufacturers must consider broadly consulting persons related to PWDs as they develop these devices and, by doing so, incorporating their views into the design considerations, which will ultimately increase their readiness to recommend the use of these devices.

Results also showed that facilitating conditions (FC) was among the constructs that explained the highest amount of variance (t-statistics = 10.351) and supported the hypothesis posited that facilitating conditions have a positive impact on the PWDs’ behavioral intention to use healthcare wearable devices. This finding is consistent with previous studies conducted by Melenhorst et al., 2006, Alvseike & Br nick 2012, and Czaja et al., 2006, who suggested that facilitating conditions, such as an individual’s intellectual and cognitive abilities and perceived cost of learning new technologies, affect the adoption of technology [46,79,80,81,82]. Facilitating conditions in the actual use of healthcare wearable devices may be influenced by the ratio of device prices and disposable income, device capabilities, and technical know-how on the part of caregivers, patients, and medical practitioners. Most caregivers in developing economies are either living below the acceptable minimum wage or are making barely enough income to be able to dispose of excess to procure healthcare wearable devices for their relatives. Hence, there is the need for stakeholders to consider investments into strategies that will facilitate the use of wearable devices. Healthcare costs should be remodeled to focus on reducing costs and increasing access to these devices.

Despite the positive receptiveness of both patients and caregivers to healthcare wearable devices/technology adoption, some studies have recorded evidence of negative attitudes toward healthcare wearable device adoption. In their study on the awareness, requirements, and barriers to the use of assistive technology designed to enable independence of people suffering from dementia, van den Heuvel et al. 2012 observed that the elderly are reluctant to accept and use new technology [83]. This position is consistent with our current study, where resistance to change (RC) was found to be significant and had a negative influence on behavioral intention to use healthcare wearable devices, as posited. This is also in line with the position expressed by most researchers that elderly people have a high degree of resistance to change [63] and, since dementia is commonly known to be associated with aging, it is expected that older adults’ resistance to change will impact their actual behavior of healthcare wearable devices. That notwithstanding, this negative attitude towards actual use might vary according to the severity or the stage of the patients and, thus, may not be so representative of the broad spectrum of dementia patients. Studies have also revealed that patients may decline the use of technology due to concerns about the unsuitability of technologies [84,85]. The reason PWDs may portray a negative attitude towards the actual use of healthcare wearable devices could be due to mismatched expectations and need satisfaction of what they think and perceive healthcare wearable devices should and can do for them [86,87,88]. Thus, there is the need to regulate these expectations by intensifying health campaigns and education targeted at increasing the use of wearable devices. Institutions, such as the Foods and Drugs Authority (FDA) and the standards authority (GSA), need to implement and strengthen frameworks that will focus on eliminating exaggerated claims by advertisement companies of what healthcare wearable devices should and can do for PWDs.

This study further observed that technology anxiety was significant but did not negatively impact the actual use behavior of healthcare wearable devices. Though the effect of technology anxiety was not negative, attention needs to be given to the role it could play in reducing the use of healthcare wearable devices, which will consequently impact the quality of life of PWDs. Due to the burden associated with the use of technology, such as frustration, confusion, discomfort, embarrassment, or anxiety [77,87,89], most PWDs or caregivers may not be interested in using or recommending the use of healthcare wearable devices. Not only does technology burden overwhelm PWDs, but research has also shown caregivers tend to exhibit negative attitudes towards the use of technology as simple as system reminders used by caregivers to continuously remind patients to use devices [90,91,92]. The use of technology is also perceived to cause discomfort [83,84], fear of becoming overly dependent on technology by patients, and concerns of caregivers that they may be replaced with machines [93]; thus, all these concerns need to be alleviated by stakeholders in the healthcare sector to scale up the actual use behavior of these devices.

A common issue that comes to mind regarding the use of healthcare wearable devices is the ethical grey areas. Issues regarding privacy and confidentiality: “how are my privacy and confidentiality protected?”, and autonomy: “ability to give full consent and data ownership” are most often seen to contribute greatly to the decision-making process of whether or not to use healthcare technologies [94]. Similarly, perceived fear of PWDs that they are been constantly monitored tend to restrict their actual use behavior [95]. Hence, it was imperative to preserve the autonomy of patients, as the loss of personal freedom can lead to infantilization [96].

In terms of the relationship between behavioral intention and actual use behavior of healthcare wearable devices, this study identified a positive relationship between the two constructs. Adapting from the literature of Carlsson et al. 2006 [97], this study asserts that the higher the behavioral intention to adopt healthcare wearable devices is, the greater the actual use behavior of such devices, which eventually impacts the quality of life of dementia patients.

Undertaking research in dementia and other neurological degenerative diseases is complex and poses several challenges [98,99]. To the best of our knowledge, no other study has empirically assessed the impact of actual use behavior of healthcare wearable devices on the quality of life of PWDs in the context of developing countries using extended UTAUT and SF-36 instruments, thus making the insights from this study very useful in guiding future research in this area. Despite the strengths of this study, there were some limitations that could be explored in future studies.

Firstly, the study was limited to the geographical analysis of caregivers and PWDs in three regions of Ghana. Although some respondents were from other locations, the majority lived in Greater Accra, Ghana’s capital city, due to its closeness to the researchers and the high expense of traveling to other places. Thus, future studies could consider extending by uniformly dispersing the sample demographics over all administrative regions of Ghana to offer a larger view of the actual use behavior of healthcare wearable devices and their implications on the quality of life of PWDs in Ghana.

Secondly, though studies have shown dementia is associated with advanced aging, others have also found an increasing trend in the early onset of dementia (EOD) [100]. However, this study limited the recruitment of PWDs to 50 years and above, excluding respondents who could potentially have an early onset of dementia. Thus, future studies could investigate the impact of healthcare wearable device use on quality of life among people with early onset of dementia in order to have a comparative understanding of the similarities or differences in the predictors and the overall impact of the use of these devices between EODs and older adults.

Finally, though this current study did not delve into the specific devices preferred and how widely they are used in Ghana, it will be beneficial for future studies to explore these themes to guide future discussions and stakeholders in this area in their effort to increase adoption and improve healthcare interventions for PWDS.

## 6. Conclusions

Based on these findings, this study concludes that the actual use behavior of healthcare wearable devices impacts the quality of life of dementia patients. The significant relationship between FC and BI suggests that, to reduce the burden of dementia for PWDs and caregivers, improve the quality of life, and ultimately promote independent living amongst PWDs, there is the need to invest more into the adoption of these devices for PWDs by either making them freely available or at a subsidized cost. Policy makers must also make sure they create the necessary conditions that will facilitate the use of these devices, for example, making less conspicuous devices, as this has shown to significantly reduce elders’ anxiety about technology use. They must create more awareness of measures being implemented to safeguard patients’ data and to reduce risks associated with the use of these devices. Furthermore, there is the need to also provide reliable and sustainable technical support to caregivers and medical practitioners who help in monitoring the smooth use of these healthcare wearable devices to allay their fears concerning hacking and data theft.

As this study has found a moderating effect of resistance to change and social influence on behavioral intention, it is suggested that attention be given to the factors that could cause PWDs to resist the use of these devices, such as the fear that the use of healthcare wearable devices will interfere with the way they deal with relevant health problems and the fear that the use of healthcare wearable devices will change the way PWDs interact with other people.

Finally, it is suggested that caregivers—relatives and medical practitioners—be involved in the decision-making process when policy makers plan to roll out interventions related to the adoption of healthcare wearable devices, as their perception (social influence) plays a key role in the actual use behavior of these devices.

## Figures and Tables

**Table 1:** Sample demographic characteristics (*n* = 320).

Variable		Caregivers	PWDs
Age (years)			
	18 years	4 (7%)	0 (0%)
	19 to 31	13 (22%)	0 (0%)
	32 to 44	19 (33%)	0 (0%)
	45 to 54	14 (24%)	75 (28%)
	55 to 64	6 (10%)	120 (46%)
	65 to 74	2 (3%)	54 (21%)
	75 and over	0 (0%)	13 (5%)
Gender, *n* (%)			
	Female	32 (55%)	143 (55%)
	Male	26 (45%)	119 (45%)
Marital Status			
	Single	33 (57%)	44 (17%)
	Married	12 (21%)	121 (46%)
	Widowed	0 (0%)	25 (10%)
	Widower	0 (0%)	23 (9%)
	Divorced	13 (22%)	49 (19%)
Level of Education			
	No Formal Education	3 (5%)	19 (7%)
	Primary School	4 (7%)	56 (21%)
	Less Than JHS	1 (2%)	2 (1%)
	Junior High School (JHS)	7 (12%)	82 (31%)
	Less Than SHS	15 (26%)	15 (6%)
	Senior High School (SHS)	3 (5%)	37 (14%)
	Nursing Diploma	5 (9%)	17 (6%)
	Teacher College Diploma	7 (12%)	16 (6%)
	Higher National Diploma (HND)	1 (2%)	7 (3%)
	Bachelor’s Degree	12 (21%)	11 (4%)
Region of Residence			
	Greater Accra	25 (43%)	112 (43%)
	Eastern	14 (24%)	34 (26%)
	Ashanti	19 (33%)	68 (32%)
Years of Illness or Caregiving			
	Less than 1 year	10 (17%)	23 (9%)
	1 to 2 years	19 (33%)	52 (20%)
	3 to 4 years	8 (14%)	109 (42%)
	5 to 6 years	11 (19%)	55 (21%)
	7 to 8 years	6 (10%)	11 (4%)
	9 to 10 years	3 (5%)	11 (4%)
	Over 20 years	1 (2%)	1 (0%)
MMSE Score		**Frequency**	**Percentage**
	Mild	14	4%
	Moderate	248	78%
	Severe	58	18%

Note: due to rounding errors, column-wise percentages may not equal 100%.

**Table 2:** Reliability, central tendency, and variability of the SF-36 scales.

Scale	Cronbach Alpha	Cronbach Alpha Based on Standardized Items	*n* of Items	Mean	SD
Physical Functioning	0.82	0.82	10	886.875	134.022
Role Physical	0.70	0.70	4	332.81	108.067
Bodily Pain	0.70	0.70	2	187.063	20.128
General Health	0.63	0.64	5	382.578	77.171
Vitality	0.70	0.70	4	332.813	108.068
Social Functioning	0.70	0.70	2	186.938	20.172
Role Emotion	0.60	0.60	3	274.375	62.595
Mental Health	0.70	0.68	5	304.156	61.489

**Table 3:** Measurement model.

Constructs	Notation	Convergent Validity		Internal Consistency Reliability	
		Outer Loading	AVE	α	CR
Behavioral Intention	BI1	0.878	0.764	0.85	0.91
	BI2	0.865			
	BI3	0.879			
Effort Expectancy	EE1	0.778	0.661	0.83	0.89
	EE2	0.822			
	EE3	0.873			
	EE4	0.776			
Facilitating Conditions	FC1	0.743	0.580	0.76	0.85
	FC2	0.785			
	FC3	0.768			
	FC4	0.749			
Performance Expectancy	PE1	0.896	0.756	0.84	0.90
	PE2	0.896			
	PE3	0.813			
Resistance to Change	RC1	0.844	0.725	0.87	0.91
	RC2	0.842			
	RC3	0.871			
	RC4	0.849			
Social Influence	SI1	0.757	0.681	0.77	0.87
	SI3	0.824			
	SI4	0.890			
Technology Anxiety	TA1	0.741	0.603	0.87	0.90
	TA2	0.720			
	TA3	0.694			
	TA5	0.805			
	TA6	0.837			
	TA7	0.849			
Actual Use Behavior	AUB1	0.745	0.62	0.75	0.85
	AUB2	0.820			
	AUB3	0.871			
Quality of Life	PH	0.854	0.682	0.54	0.81
	MT	0.797			

Abbreviations: Average of variance extracted (AVE), Composite reliability (CR), Cronbach alpha (α).

**Table 4:** Structural model.

Hypothesis	Path	*β*		t-Statistics	*p*-Value	Hypothesis Supported or Not
H1	PE ^a^→ BI ^b^	0.052		1.145	0.25	Not Supported
H2	EE ^c^→ BI	0.295		4.410	<0.001	Supported
H3	SI ^d^→ BI	0.253		4.929	<0.001	Supported
H4	FC ^e^→ BI	0.366		5.383	<0.001	Supported
H5	FC→AUB ^f^	0.524		10.351	<0.001	Supported
H6a	BI→AUB	0.301		5.666	<0.001	Supported
H7	RC ^h^→BI	−0.170		2.366	0.01	Supported
H8	TA ^i^→ BI	0.206		2.912	<0.001	Supported
H9	AUB→QoL ^g^	0.639		17.448	<0.001	Supported
Result of the Coefficients of Determination (*R*^2^)						
CONSTRUCTS	*R* Square		*R* Square Adjusted		Remarks	
AUB	0.572		0.569		Moderate	
BI	0.560		0.551		Moderate	
QoL	0.408		0.406		Moderate	
Result of Blindfolding and Predictive Relevance (*Q^2^*) and Effect Size (*q*^2^)						
CONSTRUCTS	*Q* ^2^		CONSTRUCTS		*q* ^2^	
AUB	0.370		AUB→QoL		0.157	
BI	0.407		BI→AUB		0.062	
QoL	0.271					

Abbreviations: ^a^ PE, performance expectancy; ^b^ BI, behavioral intention; ^c^ EE, effort expectancy; ^d^ SI, social influence; ^e^ FC, facilitating conditions; ^f^ AUB, actual use behavior; ^g^ QoL, quality of life; ^h^ RC, resistance to change; ^i^ TA, technology anxiety.

**Table 5:** Result of mediation.

Indirect Effects						
	Original Sample (O)	Sample Mean (M)	Standard Deviation (STDEV)	t Statistics (|O/STDEV|)	*p* Value	Remark Significant?
BI→AUB						
FC→AUB	0.110	0.110	0.032	3.442	<0.001	Yes
FC→BI						
Direct Effects						
BI→AUB	0.301	0.299	0.053	5.666	<0.001	Yes
FC→AUB	0.524	0.525	0.051	10.351	<0.001	Yes
FC→BI	0.366	0.364	0.068	5.383	<0.001	Yes

**Table 6:** Bootstrapping results for the moderating effects.

Hypothesis	Path	*β*	t-Statistics	*p* Value	Remarks
H10a	RC × EE→BI	0.239	1.349	0.18	Not Supported
H10b	RC × SI→BI	−0.255	2.383	0.01	Supported
H10c	RC × FC→BI	−0.214	1.310	0.19	Not Supported
H11a	TA × EE→BI	−0.052	0.312	0.76	Not Supported
H11b	TA × SI→BI	0.093	0.950	0.34	Not Supported
H11c	TA × FC→BI	0.168	1.071	0.29	Not Supported

## Data Availability

Data for this study are available on request from the corresponding author and first author.

## Appendix A

**Table A1:** Measurement instruments related to the extended UTAUT model.

Constructs	Items	Descriptions	Literature Sources
Behavioral Intention	BI1	I/patient intend(s) to use healthcare wearable devices in the near future	[2,101,102]
	BI2	I/patient intend(s) intend to use healthcare wearable devices at every opportunity in the future	
	BI3	I/patient plan(s) to increase his/her/my use of healthcare wearable devices in the future	
Effort Expectancy	EE1	Learning how to use healthcare wearable devices will be easy for me/patient	[2,13,103]
	EE2	I/patient find(s) healthcare wearable devices easy to use	
	EE3	It is easy for me/patient to become skillful at using healthcare wearable devices	
	EE4	My/patient’s interaction with healthcare wearable devices is clear and understandable	
Facilitating Conditions	FC1	I/patient have/has the necessary resources to use healthcare wearable devices	[2,103]
	FC2	I/patient have/has the knowledge necessary to use healthcare wearable devices	
	FC3	I/patient know my/his/her medical practitioners have the necessary knowledge to help me/him/her use healthcare wearable devices	
	FC4	Healthcare wearable devices are compatible with other technologies the I/patient use(s)	
Performance Expectancy	PE1	I/patient find(s) the healthcare wearable device useful in my/his/her live	[2,13,39,104]
	PE2	Using healthcare wearable device will help me/patient accomplish things more quickly	
	PE3	Using healthcare wearable device will improve my/patient’s quality of daily healthcare	
Resistance to Change	RC1	I/patient don’t want healthcare wearable devices to interfere with the way I/he/she deal(s) with relevant health problems	[2,13,48,103]
	RC2	I/patient don’t/doesn’t want healthcare wearable devices to change the way I/she/he lived my/his/her life before	
	RC3	I/patient don’t/doesn’t want healthcare wearable devices to change the way I/he/she interact (s) with other people	
	RC4	I/patient don’t/doesn’t want healthcare wearable devices to change the way my/his/her medical practitioners handle(s) my/his/her health issues	
Social Influence	SI1	People who are important to me/patient think that I/he/she should use healthcare wearable devices	[13,103,104]
	SI2	People who influence me/patient think I/she/he should use healthcare wearable devices	
	SI3	People whose opinions are valuable to me/patient would prefer that I/he/she use(s) healthcare wearable devices	
	SI4	Medical Practitioners think using healthcare wearable devices will enhance my/patient’s quality of life	
Technology Anxiety	TA1	Wearing healthcare wearable devices would make me/patient nervous	[2,12]
	TA2	Using healthcare wearable devices make me/patient worried	
	TA3	Using healthcare wearable devices may make me/patient to be confuse	
	TA4	Using healthcare wearable devices make me/patient feel uncomfortable	
	TA5	Using healthcare wearable devices may cause me/patient to perceive increase in the chances of me/him/her losing my/his/her cognitive abilities completely	
	TA6	Using healthcare wearable devices make me/patient feel less human	
	TA7	Using healthcare wearable devices make me/patient feel unsafe due to security issues such as hacking	
Actual Use Behavior	UB1	Using healthcare wearable devices seem to be a pleasant experience for me/patient	[2,105]
	UB2	I/patient use(s) healthcare wearable devices currently	
	UB3	I/patient intend(s) to spend time on the use of healthcare wearable devices	

**Table A2:** Questionnaire items, frequency, and percentage distributions of modified items related to the quality of life (QoL), adapted from Ware 1993′s SF-36.

Physical Functioning	Yes, Limited a Lot					Yes, Limited a Little						Not Limited at All			
	f			%		f				%		f			%
Healthcare wearable devices limit (patient/me) in doing vigorous activities	11			3.4		43				13.4		266			83.1
Healthcare wearable devices limit (patient/me) in doing moderate activities.						97				30.3		223			69.7
Healthcare wearable devices limit (patient/me) in lifting or carrying groceries						77				24.1		243			75.9
Healthcare wearable devices limit (patient/me) in climbing several flights of stairs	1			0.3		60				18.8		259			80.9
Healthcare wearable devices limit (patient/me) in climbing one flight of stairs	2			0.6		82				25.6		236			73.8
Healthcare wearable devices limit (patient/me) in bending, kneeling, or stooping	1			0.3		54				16.9		265			82.8
Healthcare wearable devices limit (patient/me) in walking more than a mile	4			1.3		67				20.9		249			77.8
Healthcare wearable devices limit (patient/me) in walking several hundred yards						62				19.4		258			80.6
Healthcare wearable devices limit (patient/me) in walking one hundred yards	1			0.3		62				19.4		257			80.3
Healthcare wearable devices limit (patient/me) in bathing or dressing him/herself	4			1.3		72				22.5		244			76.3
**Role-Physical**	**Yes**									**No**					
	**f**					**%**				**f**					**%**
Healthcare wearable devices help (patient/me) in cutting down the amount of time (patient/I) spent on work or other activities	61					19.1				259					80.9
Healthcare wearable devices make (patient/me) accomplish less than (patient/I) would like	47					14.7				273					85.3
Healthcare wearable devices make (patient/me) limited in his/her/my kind of work or other activities	62					19.4				258					80.6
Healthcare wearable devices make (patient/me) have difficulties performing work or other activities (e.g., it took extra effort)	45					14.1				275					85.9
**Bodily Pain**	**None**					**Very mild**				**Mild**					
	**f**		**%**			**f**		**%**		**f**					**%**
How much bodily pain have you (patient) had during the past 4 weeks while using healthcare wearable devices?	194		60.6			97		30.3		29					9.1
	**Not at all**					**Not at all**							**A little bit**		
	**f**		**%**			**f**				**%**			**f**		**%**
During the past 4 weeks, how much did pain interfere with your normal work since using healthcare wearable devices?	284		88.8			20				6.3			16		5.0
**General Health**	**Definitely True**		**Mostly True**			**Don’t Know**				**Mostly False**			**Definitely False**		
	**f**	**%**	**f**		**%**	**f**			**%**	**f**	**%**		**f**		**%**
With healthcare wearable devices, it seem (patient/I) get sick a little easier than other people	12	3.8	14		4.4					268	83.8		26		8.1
With healthcare wearable devices, (patient/I) am(is) as healthy as anybody (patient/I) know	201	62.8				70			21.9	49	15.3				
With Healthcare wearable devices, (patient/I) expect(s) (patient’s/my) health to get worse	11	3.4	45		14.1	19			5.9	238	74.4		7		2.2
With Healthcare wearable devices (patient’s/my) health is excellent	268	83.8				8			2.5	44	13.8				
	**Excellent**		**Very Good**			**Good**				**Fair**			**Poor**		
	**f**	**%**	**f**		**%**	**f**			**%**	**f**	**%**		**f**		**%**
With the help of healthcare wearable devices, (patient/I) will say (his/her/my) health is:	125	39.1	167		52.2					24	7.5		4		1.3
**Vitality**	**All of the time**								**None of the time**						
	**f**		**%**						**f**		**%**				
With healthcare wearable devices, (patient’s/I) feel full of life	259		80.9						61		19.1				
With healthcare wearable devices, (patient’s/I) have a lot of energy	273		85.3						47		14.7				
With healthcare wearable devices, (patient’s/I) feel worn out	62		19.4						258		80.6				
With healthcare wearable devices, (patient’s/I) feel tired	45		14.1						275		85.9				
**Role-Emotional**	**Yes**								**No**						
	**f**		**%**						**f**		**%**				
Using healthcare wearable devices has made me (patient) cut down the amount of time spent on work or other activities	293		91.6						27		8.4				
Using healthcare wearable devices has made (patient/me) accomplished less than (patient/I) would have liked	26		8.1						294		91.9				
Using healthcare wearable devices made (patient/me) do work or other activities less carefully than usual	29		9.1						291		90.9				
**Mental Health**	**All of the time**		**Some of the time**						**A little of the time**		**None of the time**				
	**f**	**%**	**f**			**%**			**f**	**%**	**f**			**%**	
Using healthcare wearable devices has made (patient/me) to be a very nervous person	4	1.3	22			6.9			95	29.7	199			62.2	
Using healthcare wearable devices has made (patient/me) feel so down in the dumps that nothing could cheer (him/her/me) up	2	0.6	23			7.2			129	40.3	166			51.9	
Using healthcare wearable devices has made (patient/me) felt calm and peaceful	133	41.6	148			46.3			38	11.9	1			0.3	
Using healthcare wearable devices has made (patient/me) felt downhearted and depressed	3	0.9	21			6.6			145	45.3	151			47.2	
Using healthcare wearable devices has made (patient/me) to be happy	132	42.2	158			49.4			26	8.1	1			0.3	
**Reported Health** **Transition**	**Much better now than one year ago**		**Somewhat better now than one year ago**			**About the same as one year ago**			**Somewhat worse now than one year ago**		**Much worse now than one year ago**				
	**f**	**%**	**f**		**%**	**f**	**%**		**f**	**%**	**f**			**%**	
How is your (patient’s) health now compared to 1 year ago since using healthcare wearable devices?	14	4.4	136		42.5	126	39.4		30	9.4	14			4.4	
**Social Functioning**	**Not at all**				**Slightly**					**Moderately**					
	**f**	**%**			**f**		**%**			**f**				**%**	
During the past 4 weeks since using healthcare wearable devices, to what extent has your (patient’s) physical health or emotional problems interfered with your normal social activities with family, friends, neighbors, or groups?	194	60.6			97		30.3			29				9.1	
	**Some of the time**				**A little of the time**					**None of the time**					
	**f**	**%**			**f**		**%**			**f**				**%**	
During the past 4 weeks since using healthcare wearable devices, how much of the time has your physical health or emotional problems interfered with your social activities (like visiting with friends, relatives, etc.)?	17	5.3			20		6.3			283				88.4	

**Table A3:** Discriminant validity.

Correlation Matrix and Square Root of the AVE Assessed by Fornell–Larcker Criterion									
	AUB	BI	EE	FC	PE	QoL	RC	SI	TA
AUB	0.814								
BI	0.644	0.874							
EE	0.543	0.647	0.813						
FC	0.721	0.654	0.856	0.762					
PE	0.185	0.249	0.086	0.050	0.869				
QoL	0.639	0.813	0.640	0.802	0.195	0.826			
RC	0.237	0.193	0.235	0.280	0.162	0.205	**0.852**		
SI	0.121	0.385	0.144	0.117	0.577	0.242	0.056	**0.825**	
TA	0.219	0.251	0.182	0.246	0.168	0.263	0.819	0.125	**0.777**
Heterotrait–Monotrait Ratio of correlation (HTMT)									
	AUB	BI	EE	FC	PE	QoL	RC	SI	TA
AUB									
BI	0.802								
EE	0.673	0.764							
FC	0.933	0.808	1.073						
PE	0.241	0.279	0.137	0.127					
QoL	0.998	1.178	0.949	1.276	0.297				
RC	0.294	0.221	0.269	0.339	0.195	0.299			
SI	0.177	0.456	0.183	0.172	0.703	0.336	0.096		
TA	0.279	0.280	0.215	0.309	0.208	0.384	0.972	0.146	

**Table A4:** Cross loadings.

	AUB	BI	EE	FC	PE	QoL	RC	SI	TA
AUB1	0.745	0.418	0.381	0.499	0.254	0.430	0.213	0.098	0.153
AUB2	0.820	0.543	0.450	0.607	0.130	0.549	0.191	0.151	0.190
AUB3	0.871	0.593	0.486	0.642	0.094	0.568	0.184	0.052	0.188
BI1	0.565	0.878	0.577	0.595	0.298	0.854	0.166	0.321	0.191
BI2	0.559	0.865	0.605	0.581	0.225	0.618	0.214	0.331	0.276
BI3	0.565	0.879	0.514	0.538	0.126	0.659	0.124	0.359	0.189
EE1	0.382	0.477	0.778	0.576	0.003	0.491	0.106	0.044	0.118
EE2	0.359	0.503	0.822	0.667	0.121	0.523	0.167	0.103	0.099
EE3	0.407	0.505	0.873	0.768	−0.029	0.501	0.229	0.093	0.164
EE4	0.583	0.598	0.776	0.749	0.162	0.554	0.244	0.209	0.199
FC1	0.487	0.435	0.475	0.743	0.003	0.797	0.175	0.061	0.247
FC2	0.680	0.439	0.490	0.785	−0.008	0.605	0.201	−0.015	0.144
FC3	0.407	0.505	0.873	0.768	−0.029	0.501	0.229	0.093	0.164
FC4	0.583	0.598	0.776	0.749	0.162	0.554	0.244	0.209	0.199
MT	0.487	0.435	0.475	0.743	0.003	0.797	0.175	0.061	0.247
PE1	0.160	0.250	0.065	0.066	0.896	0.203	0.141	0.572	0.143
PE2	0.186	0.233	0.096	0.027	0.896	0.166	0.123	0.489	0.137
PE3	0.126	0.138	0.059	0.034	0.813	0.123	0.176	0.417	0.176
PH	0.565	0.878	0.577	0.595	0.298	0.854	0.166	0.321	0.191
RC1	0.222	0.149	0.169	0.197	0.165	0.171	0.844	0.041	0.720
RC2	0.163	0.160	0.209	0.218	0.125	0.153	0.842	0.059	0.694
RC3	0.171	0.148	0.219	0.265	0.116	0.177	0.871	0.045	0.652
RC4	0.243	0.192	0.200	0.268	0.143	0.194	0.849	0.045	0.717
SI1	0.030	0.218	0.150	0.099	0.455	0.103	0.025	0.757	0.064
SI3	0.162	0.304	0.108	0.130	0.505	0.198	0.144	0.824	0.187
SI4	0.094	0.393	0.115	0.072	0.481	0.262	−0.017	0.890	0.064
TA1	0.216	0.178	0.129	0.274	0.104	0.302	0.546	0.102	0.741
TA2	0.222	0.149	0.169	0.197	0.165	0.171	0.844	0.041	0.720
TA3	0.163	0.160	0.209	0.218	0.125	0.153	0.842	0.059	0.694
TA5	0.156	0.146	0.097	0.129	0.144	0.157	0.604	0.061	0.805
TA6	0.114	0.233	0.153	0.194	0.145	0.229	0.565	0.147	0.837
TA7	0.178	0.256	0.114	0.153	0.118	0.200	0.561	0.130	0.849

**Table A5:** Collinearity statistics (VIF).

	AUB	BI	EE	FC	PE	QoL	RC	SI	TA
AUB						1.000			
BI	1.747								
EE		3.814							
FC	1.747	3.892							
PE		1.548							
QoL									
RC		3.187							
SI		1.555							
TA		3.127							
